# Factors associated with occupational asthma among food industry workers: A systematic review

**DOI:** 10.1371/journal.pone.0287040

**Published:** 2023-06-12

**Authors:** Ahmed Syahmi Syafiq Md Zamri, Muhammad Zulhilmie Saruddin, Amin Harun, Siti Fatimah Abd. Aziz, Abi Khairul Aizad Za’bah, Rahmat Dapari, Mohd Rohaizat Hassan, Nazri Che Dom, Syed Sharizman Syed Abdul Rahim

**Affiliations:** 1 Department of Community Health, Faculty of Medicine and Health Sciences, Universiti Putra Malaysia, Serdang, Selangor, Malaysia; 2 Department of Community Health, Faculty of Medicine, National University of Malaysia, Kuala Lumpur, Malaysia; 3 Faculty of Health Sciences, Universiti Teknologi MARA, Puncak Alam, Selangor, Malaysia; 4 Public Health Medicine Department, Faculty of Medicine and Health Sciences, Universiti Malaysia Sabah, Kota Kinabalu, Sabah, Malaysia; Satyawati College, University of Delhi, INDIA

## Abstract

**Introduction:**

Occupational asthma (OA) is a type of Work-Related Asthma characterised by variable airflow limitation and/or inflammation due to causes and conditions attributable to a particular occupational environment, and not to stimuli encountered outside the workplace. There is an increasing need to extend the depth of knowledge of OA to better manage this condition, especially among food industry workers who are affected by it.

**Objective:**

This systematic review aimed to determine the factors associated with occupational asthma among food industry workers by electronically collecting articles from two databases (Medline and Scopus).

**Methods:**

This systematic review was prepared in accordance with the PRISMA (Preferred Reporting Items for Systematic Reviews and Meta Analyses) updated guideline. Two independent reviewers screened the titles and abstracts of the collected data, which were then stored in Endnote20 based on the inclusion and exclusion criteria. The included articles have been critically appraised to assess the quality of the studies using the Mixed Methods Appraisal Tool (MMAT).

**Result:**

The search yielded 82 articles from Medline and 85 from SCOPUS, resulting in 167 unique hits. Only 22 articles have been included in the full-text assessment following a rigorous selection screening. Of the 22 articles identified, five were included in the final review. Several factors were found to have contributed to occupational asthma among food industry workers. They were classified into two categories: (1) work environment-related factors; and (2) individual factors.

**Conclusion:**

Several work environment and individual-related factors were found to be associated with OA among food industry workers. A better understanding of the development of the disease and its potential risk factors is needed because it can affect worker’s quality of life. Pre-employment and periodic medical surveillance should be conducted to assess and detect any possible risk of developing occupational asthma among workers.

## Introduction

There are approximately 17% to 25% of adult asthma cases related to Work-Related Asthma (WRA) [1, 2]. Work-Related Asthma can be further classified as occupational asthma (OA) and work exacerbated asthma. Occupational asthma (OA) is defined as a disease characterised by variable airflow limitation and/or inflammation due to causes and conditions attributable to a particular occupational environment, and not to stimuli encountered outside of the workplace. Meanwhile, work exacerbated asthma is defined as pre-existing or concurrent asthma that is worsened by workplace conditions [1]. The types of OA can be either allergic (sensitiser-induced) or non-allergic (irritant-induced). Most OA cases in the food industry are allergic cases caused by animal or vegetal high molecular weight protein. These irritants aerosolised can be inhaled in the form of dust, steam, and vapours produced at various stages of food processing. Meanwhile, known irritants can include cereals and flours (wheat, rye, buckwheat, barley), animals (farms, laboratory, and seafood), latex, and enzymes (amylase, subtilisine, maxatase, pancreatin, bromelain) [3].

Factors that can be associated with WRA are categorised into two, which are working environmental factors and Individual Susceptibility [4]. Levels of exposure to irritants, as well as the modes and routes of exposure and co-exposure to pollutants have been identified as potential environmental factors involved in the development of OA. Meanwhile, atopy, genetic factors, rhinitis, pre-existing non-specific airway hyperresponsiveness, and gender are factors that predispose workers to OA, even though they might share the same exposures.

Most patients who developed OA eventually progressed to chronic asthma. It was reported that one in six patients meets the criteria for severe asthma [4]. With the progress of the disease, patients with OA are prone to develop anxiety, depression, and have an impaired quality of life, as the result of both long-term illness and poor socioeconomic outcome [5–7]. In order to completely avoid their allergens, patients will have to either adapt or change their workplaces or worse, quit their job that could result in loss of productivity and unemployment [6].

Currently, knowledge of OA is growing, especially in clinical phenotypes, biomarkers, diagnosis, and management strategies [8]. However, the number of irritants in food industries is also growing, alongside technology and development progress [9]. Management of OA in food industries must include workplace control measure, as well as specific pharmacological therapy. Specific immunological therapy has been demonstrated as showing promising results of reducing OA symptoms among affected workers, even when they have returned to work [3]. Hence, this review aimed to determine the associated factors that may currently be affecting the development of OA among food industry workers.

## Methods

This systematic review was prepared in accordance with the PRISMA (Preferred Reporting Items for Systematic Reviews and Meta Analyses) updated guideline. The objective of this review was to identify the factors associated with occupational asthma among food industry workers. The components of mnemonic PEO (population, exposure, outcome) have been established as follows:

- Population: food industry workers.
- Exposure: factors associated with occupational asthma
- Outcome: occupational asthma.

### Searching strategy

The literature search was conducted from 1 November 2022 to 30 November 2022 using Medline and Scopus databases. The following keywords were used to search for related articles: “factor*” OR “determinant*” AND "occupational asthma" OR "job asthma" OR "respiratory allergy" AND "food industry" OR "food worker" OR "food handler". All retrieved articles were imported into EndNote20 library.

### Eligibility criteria

The inclusion criteria were as follows: (1) published in the English language; (2) original articles that included cohort, case-control, and cross-sectional studies investigating the associated factors of occupational asthma; (3) study population that included food industry workers; and (4) occupational asthma or work-related chronic respiratory symptoms as an outcome of the study. In contrast, mixed methods and qualitative studies, as well as non-original articles, such as conference proceedings, perspectives, commentaries, opinions, reports, systematic reviews, and meta-analyses were excluded. The publication period was decided to start from 2010 onwards.

### Study selection

Two independent reviewers screened the titles and abstracts of the retrieved materials against the inclusion and exclusion criteria. Potential articles that were identified during the main screening were kept, and the full texts were independently reviewed by five independent reviewers based on the inclusion and exclusion criteria. Any disagreements that arose were discussed as a group among all five reviewers.

### Critical appraisal and data extraction

Quality appraisal was conducted using the Mixed Methods Appraisal Tool (MMAT). The quality of each selected article was evaluated based on methodological criteria, which included five core quality criteria [10]. Two pairs of reviewers extracted the data, which were then independently assessed by a second pairs of reviewers. Eligible articles were analysed in detail using the content analysis method without any statistical tests.

## Results

**Fig 1:**
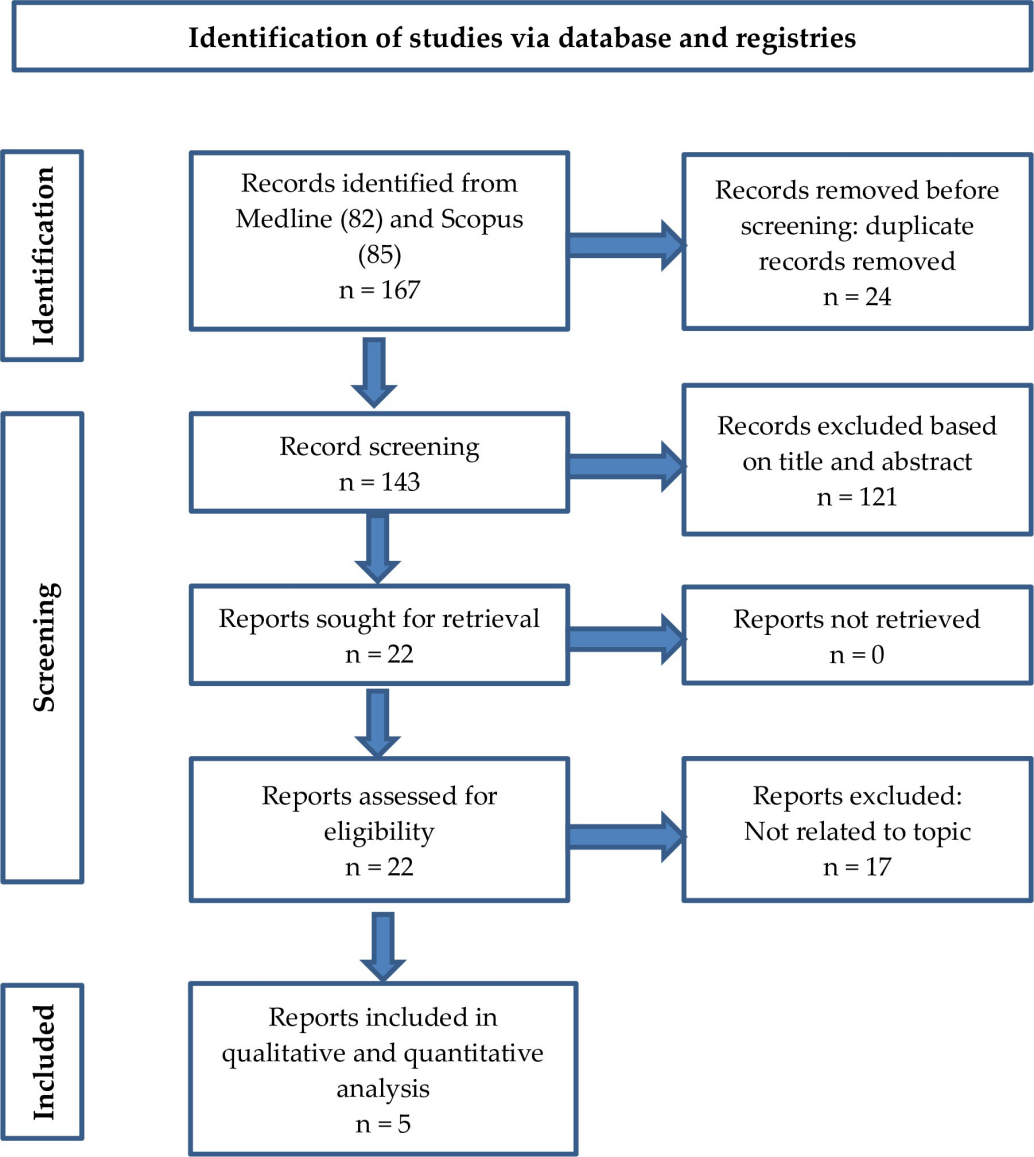
PRISMA flow diagram for the systematic review.

The search yielded 82 articles from Medline and 85 from SCOPUS, resulting in 167 unique hits. Only 22 articles have been included in the full-text assessment following a rigorous selection screening, as shown in the PRISMA flow diagram (Fig 1). Details of the selected studies in this review are presented in Table 1. The findings of five studies are included in this systematic review, as listed in Table 2. The analysed articles were published between 2013 and 2020, whereby four articles were cross-sectional studies, and one article was a longitudinal study. Initial duplicate articles were removed via the automated removal in EBSCO Medline database.

**Table 1:** Details of selected study locations and study designs.

Authors	Study Location	Study Design
Jonaid et al. (2017) [11]	Netherlands	Longitudinal study
Lagiso et al. (2020) [12]	Ethiopia	Cross-sectional
Alemseged et al. (2020) [13]	Ethiopia	Cross-sectional
Thomassen et al. (2017) [14]	Norway	Cross-sectional
van der Walt et al. (2013) [15]	South Africa	Cross-sectional

**Table 2:** Summary of accepted articles.

Author (Year)	Title	Study Design	Sample Size	Outcome / Method of assessment	Factors
Jonaid et al. 2017 [11]	Predicting Occupational Asthma and Rhinitis in Bakery Workers Referred for Clinical Evaluation	Longitudinal study	436	Occupational asthma a) Medical history Two clusters of correlated symptoms, including rhino-conjunctivitis and work-related upper respiratory symptoms. b) Serum total and specific IgE test - Serum total IgE was measured using a sandwich enzyme immunoassay (EIA) - Specific IgE for common allergens and specific IgE antibodies against wheat, rye, and α-amylase were measured using an earlier developed and modified EIA. c) Lung function test - Spirometry was performed according to the American Thoracic Society/European Respiratory Society standards - Non-specific bronchial hyper-responsiveness (NSBHR) was assessed using the methacholine challenge test according to the American Thoracic Society guidelines.	a) Self-administered Questionnaire Model • Work-related lower respiratory symptoms: (OR = 17.7, 95% CI = 4.2–74.6, p < 0.001) • Allergy (Work-related conjunctivitis–reflect allergy): (OR = 3.9, 95% CI = 2.4–6.2, p = 0.001) • Use of medication for lungs (last year): (OR = 12.0, 95% CI = 3.8–37.5, p < 0.001) b) Medical history model • Work-related lower respiratory symptoms: (OR = 4.0, 95% CI = 2.0–7.9, p = 0.001) • Allergy (Work-related conjunctivitis–reflect allergy): (OR = 3.9, 95% CI = 1.7–9.0, p < 0.001) • Use of medication for lungs (last year): (OR = 5.9, 95% CI = 2.7–12.5, p < 0.001)
Lagiso et al. 2020 [12]	Chronic Respiratory Symptoms, Lung Function and Associated Factors among Flour Mill Factory Workers in Hawassa City, Southern Ethiopia: “Comparative Cross-sectional Study”	Cross-sectional	406	Chronic respiratory symptoms A spirometry test for lung function was performed using the Ndd Easy On-PC spirometer. * Due to limited resources, only 108 out of 406 randomly selected participants (54 from the wheat mill and 54 from the soft drink plant industries) underwent a lung function test.	a) Socio demographic factors • Education status: Primary education (AOR = 5.8, 95% CI = 1.3–23.32, p < 0.05) b) Work-related factor • Working department: Mixing (AOR = 5.3, 95% CI = 1.7–16.6, p < 0.05) • Working experience: 1.6–9 years (AOR = 5.1, 95% CI = 2.1–12.5) 2. ≥ 10 years (AOR = 2.5, 95% CI = 1.0–6.1) • Working hours per day: > 8 hours (AOR = 2.4, 95% CI = 1.2–5.1)
Alemseged et al., 2020 [13]	Assessment of Chronic Respiratory Health Symptoms and Associated Factors among Flour Mill Factory Workers in Addis Ababa, Ethiopia, 2019: A Cross-Sectional Study	Cross-sectional	415	Chronic respiratory symptoms Respiratory health symptoms were assessed using an American Thoracic Society standard questionnaire, which was customised to fit the local context.	a) Socio-demographic factor • Age: 26–45 years old (AOR = 1.95, 95% CI = 1.17–3.24), > 45 years old (AOR = 12.33, 95% CI = 4.39–34.64) • Average monthly income: ≤ 1500 ETB (AOR = 1.68, 95% CI = 1.0–2.79) b) Workplace factors • History of past dust exposure: Yes (AOR = 1.86, 95% CI = 1.08–3.21) • Work experience: > 10 years (AOR = 2.58, 95% CI = 1.0–6.62) Factor associated with occupational asthma: a) Behavioural Factor c) Use of respiratory devices: Pieces of cloth (as PPE): (AOR = 0.44, 95% CI = 0.26–0.84)
Thomassen et al. 2017 [14]	Lung Function and Prevalence of Respiratory Symptoms in Norwegian Crab Processing Workers	Cross-sectional	433	Chronic respiratory symptoms A spirometry test for lung function was performed using a Spida USB (CareFusion 234 GmbH, Hoechberg, Germany).	a) Work-related respiratory symptoms • Wheezing (AOR = 2.71, 95% CI = 1.35–5.41, p = 0.01) • Shortness of breath (AOR = 2.77, 95% CI = 1.27–6.05, p = 0.005) • Prolonged cough (AOR = 2.97, 95% CI = 1.49–5.92, p = 0.002)
van der Walt et al., 2013 [15]	Work-related Allergic Respiratory Disease and Asthma in Spice Mill Workers is Associated with Inhalant Chili Pepper and Garlic Exposures	Cross-sectional	150	Occupational asthma a) Environmental sampling • Random collection of 62 full-shift (8-hour) personal airborne samples utilising the NIOSH occupational exposure sampling strategy manual. • As an inhalable dust sampling procedure, a PAS6 sampling head connected to a Gillian GilAir pump with constant-flow calibration at 2 L/min was used to collect samples. As the filter medium, glass fibre filters (Whatman GF/A, pore size of 1.0 mm, 25 mm diameter) were utilised. Each day of sampling utilised field blanks. A spirometry test for lung function was conducted according to ATS/ERS guidelines using a flow-volume Koko Spirometer. b) Immunological test • Serum analyses for common inhalants (Phadiatop test), and specific IgE for garlic and chilli pepper were conducted using the UniCAP assay procedure (Immuno-Cap 100 System; Phadia, Uppsala, Sweden) according to the manufacturer’s instructions. An ImmunoCAP value of ≥0.35 kUA per litre was considered positive. c) Fractional exhaled nitric oxide was determined prior to spirometry using ATS/ERS criteria using a hand-held portable nitric oxide sampling device (NIOX MINO Airway Inflammation Monitor, Aerocrine AB, Solna, Sweden).	Inhalable particulate dust (containing garlic and chili pepper allergens) • Airborne garlic allergen (> 0.235 μg/m^3^): Spice dust-related asthma-like symptoms (OR = 4.15, 95% CI = 1.09–15.72) • Airborne chili pepper (> 0.92 μg/m^3^): in Garlic monosensitisation (OR = 11.52, 95% CI = 1.17–113.11): Spice dust-related asthma-like symptom (OR = 3.16, 95% CI = 0.99–10.06)

OR: Odd Ratio

AOR: Adjusted Odd Ratio

FVC: Forced vital capacity

FEV1: Volume that has been exhaled at the end of the first second of forced expiration

### Occupational asthma

This article presents five studies that have focused on occupational asthma (OA) among food industry workers. OA is defined as work-related asthma, with chronic respiratory symptoms, which are demonstrated by a reduction in the lung function test via spirometry. Meanwhile, the development of one or more respiratory symptoms, such as a chronic cough, a chronic cough with sputum, chronic dyspnea, chronic wheezing, or a chronic chest infection, that endure for at least three months in a year is referred to as having chronic respiratory symptoms [12].

OA can be triggered by several factors, such as work-related respiratory symptoms, socio-demographic and work-related factors, exposure to inhalable particulate dust and allergens, allergy, and use of medication. Most studies included in this review reported significant effect to OA, while the factor that was determined to hinder OA was behavioural factor. In one study, flour mill factory workers used pieces of cloth as their personal protective equipment (PPE) during work, although none of them reported using proper respiratory devices. Nevertheless, this still served as a protective factor towards developing occupational-related respiratory symptoms [13].

### Factors associated with work-place environments

#### i. Exposure to inhalable particulate dust and allergens

Previous case series of work-related asthma reported specified allergens as the main sensitisers. According to systematic finding, a positive exposure response relationship was observed for particulate mass and allergen exposures in relation to occupational asthma. This finding was demonstrated using a sensitive novel allergen detection assay for garlic and chili pepper. Spice mill workers exposed to inhalable spice dust particulate (mean > 2 μg/m^3^) containing garlic and chili pepper allergen levels of 0.24 and 0.44 μg/m^3^, respectively, have shown an increased risk of work-related lower respiratory symptoms, allergic sensitisation, probable asthma, and obstructive lung disease. Dust particulate correlated strongly with airborne garlic and chili pepper allergens (r = 0.70). Airborne chili pepper and garlic allergen levels were also strongly correlated (r = 0.74) [15].

They also found that 8% of the workers have elevated FeNO of more than 50 parts per billion (ppb), which implied probable occupational allergic asthma. Workers were also more likely to have work-related ocular-nasal (OR = 2.40, 95% CI = 1.09–5.27) and asthma-like symptoms (OR = 4.15, 95% CI = 1.09–15.72) owing to spice dust inhalation when exposed to considerably higher (> 0.235 μg/m^3^) levels of airborne garlic allergen. Meanwhile, workers who were mono-sensitised to garlic were more likely exposed to airborne chili pepper (> 0.92 μg/m^3^) (OR = 11.52, 95% CI = 1.17–113.11). Additionally, the mono-sensitised garlic workers were demonstrated to have a strong association with spice dust-related asthma-like symptoms (OR = 3.16, 95% CI = 0.99 to 10.06), even after adjusting for host factors (age, atopy, smoking status, and reaction to spices/spicy foods) [15].

#### ii. Working conditions

Work-related factors, such as working department, working hours, and number of years in service were found to be positively associated with chronic respiratory symptoms. Based on this study, 108 flour mill workers showed a statistically significant reduction in FVC (p < 0.002), FEV1 (p < 0.001), and FEV1/FVC (p < 0.012) compared to soft-drinks factory workers. This difference might be due to the increased accumulation of dust in the respiratory system of flour mill workers associated with prolonged exposure at their workplace [12].

Workers in the cleaning, loading, and packing departments were 1.68 times more likely than those working in the mixing department to experience chronic respiratory problems (AOR = 5.3, 95% CI = 1.68–16.56) [12]. The risks of developing chronic respiratory symptoms were 5.1 and 2.5 times higher for employees with 1 and 5 years of work experience, respectively, compared to those with 6 and 9 years (AOR = 5.1, 95% CI = 2.05–12.48) and 10 or more years of work experience (AOR = 2.5, 95% CI = 1.01–6.11) [12]. Similar result was observed among flour mill factory workers, whereby those who have been working for more than 10 years showed higher odds of developing chronic respiratory symptoms (AOR = 2.58, 95% CI = 1.0–6.62) [13]. Meanwhile, workers who worked for more than 8 hours per day were 2.4 times more likely to acquire chronic respiratory symptoms than those who worked for less than 8 hours (AOR = 2.4, 95% CI = 1.16–5.10) [12].

Additionally, workers who were exposed to dust in the past faced higher possibility of suffering similar symptoms. Their likelihood of experiencing persistent chronic respiratory symptoms was 1.86 times greater than those that have not previously work in a dusty working environment (AOR = 1.86, 95% CI = 1.08–3.21) [13].

#### iii. Work-related symptoms

Work-related respiratory symptoms were observed to act as a predictor of OA. For example, researcher have successfully developed a diagnostic model for predicting baker’s asthma (BA) [11]. They demonstrated that the probability onset of BA among workers at risk of being sensitised can be estimated using the candidate items in both the self-administered questionnaire and the medical history model. The predictor item with a significant association (p < 0.05) to predicting BA risk was work-related lower respiratory symptoms (OR = 17.7, 95% CI = 4.2–74.6, p < 0.001), as observed in the self-administered questionnaire model, while the predictor item in the medical history model showed a lower odd ratio of 4.0 (95% CI = 2.0–7.9, p = 0.001) [11].

Inhalable bioaerosol from air samples generated during seafood processing was another risk of developing occupational health problems. Study showed that self-reported respiratory symptoms were greater among crab processing employees than among controls, and more among king crab workers than among edible crab workers [14]. The reported respiratory symptoms included wheezing (AOR = 2.71, 95% CI = 1.35–5.41, p = 0.01), shortness of breath (AOR = 2.77, 95% CI = 1.27–6.05, p = 0.005), and prolonged cough (AOR = 2.97, 95% CI = 1.49–5.92, p = 0.002). However, no significant difference was found in lung function measurements between crab processing workers and control [14]. Therefore, the increased respiratory symptoms reported were not reflected in the impairment of lung function values or asthma diagnose.

### Factors associated with individual factors

#### i. Socio-demographic factors

A study among 415 flour mill factory workers, with 48.9% and 18.8% were between 26 and 45 years old, and older than 45 years old, respectively found that age was significantly associated with chronic respiratory symptoms among these workers [13]. Workers aged 26 to 45 years old and older than 45 showed 1.95 and 12.33 times greater odds of suffering from chronic respiratory symptoms, respectively, than those aged 25 (AOR = 1.95, 95% CI = 1.17–3.24) and (AOR = 12.33, 95% CI = 4.39–34.64) [13].

The average monthly income of a worker was also correlated to the increased risk of developing chronic respiratory symptoms. The study showed that the workers with a monthly income of less than 1500 ETB (AOR = 1.68, 95% CI = 1.0–2.79) faced 1.68 times the likelihood of developing these symptoms compared to those with a higher income of more than 1500 ETB [13].

According to another study, workers who attained a primary level of education were significantly associated with chronic respiratory symptoms, which accounted for 73% of the sample. These workers were 5.8 times more likely to develop chronic respiratory symptoms than workers whose education was at secondary and higher levels (AOR = 5.8, 95% CI = 1.3–23.2) [12].

#### ii. Atopy

Atopy was observed to be a predictive risk of occupational asthma as demonstrated using a diagnostic model to examine the probability onset of BA [11]. Their results demonstrated that work-related conjunctivitis that reflect allergy was 3.9 times more likely to occur among workers than other candidate predictors studied (OR = 3.9, 95% CI = 2.4–6.2, p = 0.001) [11].

#### iii. The use of medication

The use of medication during the previous year was another strong predictor of disease severity. The use of medication predictor in the questionnaire model was more sensitive (OR = 12.0, 95% CI = 3.8–37.5, p < 0.001) than in the medical history model (OR = 5.9, 95% CI = 2.7–12.5, p < 0.001). Among bakers with a low sensitisation probability, the reported rate of medication use was very low [11].

### Risk of bias

This systematic review conducted a quality appraisal of all five studies using the Mixed Methods Appraisal Tool (MMAT). The methodology quality of these quantitative non-randomised studies was appraised using five criteria [10]. Details of the MMAT assessment for the selected studies are reported in Table 3.

**Table 3:** Details of the MMAT assessment.

Author	Type of Study	1.1 Are the participants representative of the target population?	1.2 Are measurements appropriate regarding both the outcome and intervention (or exposure)?	1.3 Are there complete outcome data?	1.4 Are the confounders accounted for in the design and analysis?	1.5 During the study period, is the intervention administered (or exposure occurred) as intended?
Jonaid et al. (2017)	Quantitative non-randomised	Yes	Yes	No	No	No
Lagiso et al. (2020)	Quantitative non-randomised	Yes	Yes	Yes	No	No
Thomassen et al. (2017)	Quantitative non-randomised	Yes	Yes	Yes	No	No
Van Der Walt et al. (2013)	Quantitative non-randomised	Yes	Yes	Yes	Yes	No
Alemseged et al. (2020)	Quantitative non-randomised	Yes	Yes	Yes	No	No

## Discussion

Several factors were found to have contributed to occupational asthma among food industry workers. They were classified into two categories: (1) work environment-related factors; and (2) individual factors.

### Work environment-related factors

Poor ventilation systems may contribute to OA due to the accumulation of dust and bioaerosol components in the air from the various work processes, which can cause adverse effects on the respiratory system [16]. Those who worked without a proper ventilation system were found to suffer higher risk of developing a respiratory illness compared to those who worked with a proper ventilation system (AOR = 2.05; 95% CI = 1. 18–3. 56) [17]. A study from Norway reported that a poor ventilation system not only exposed seafood processing workers on-board a factory vessel to the bioaerosol, but also to noxious gas [18]. In a study on bakeries in Taiwan, the indoor air qualities among the selected study sites were higher, with the Total Volatile Organic Compound (TVOC) ranging from 150–8678 ppb, than the standard level of Taiwan Air Quality (IAQ) act of 560 ppb. This study also found that a bakery with no ventilation has a higher peak concentration of inhalable flour dust in the oven and baking areas compared to a bakery with a local exhaust ventilation in the same areas. Thus, a local exhaust ventilation system must be installed to reduce the exposure concentration in the air [19]. However, this study was excluded from both the final review and Table 2 because it did not satisfy the inclusion and exclusion criteria of the current systematic review.

Another factor that has contributed to OA was the lack of training on safety and health. In a case-control study, a group of mill workers showed a higher prevalence of OA (63.9%) compared to a control group of office workers (20.7%) due to their prolonged exposure to flour dust. Based on their observation, these milling plants were not in compliance with the control and technology standards, as they have poor air quality and housekeeping procedures. Most workers were not provided with appropriate personal protective equipment (PPE), while those who were provided with PPE did not wear it properly [17]. Another study among bakers in Ghana found that the respondents, who mostly have low education, implemented their own coping mechanism to prevent inhalation exposure during work by using handkerchiefs to avoid flour dust. Although they were aware of the hazard, they were still exposed due to failure to follow the laws and regulation of health and safety standards in Ghana [20]. However, this study was not included in the final review as well as Table 2 because it did not meet the inclusion and exclusion criteria of the present systematic review.

Prolonged exposure and symptoms may lead to serious implications on morbidity-like progression into chronic respiratory illnesses, which might subsequently cause work disabling asthma, increase absenteeism, and reduce the productivity of the workers. Consequently, these illnesses may seriously impact their psychological health. Further implication on the economy may occur due to the high burden of resources on the occupational health service, and additional cost to the organisation (employer) related to treatment of workers and loss of productivity [21]. Additionally, mortality from asthma due to occupational exposure was reported at 9.9% globally, with death at the age of 15 to 79 years old and higher among the elderly. However, a study reported that the burden of death has spread to younger ages. The highest numbers of deaths mostly occurred in low- and middle-income countries in South Asia and Southeast Asia [22].

### Individual factors

Despite similar workplace exposures, it was observed that only a few workers developed OA, which strongly suggested that individual factors also play a role in the outcome of developing OA [23]. The individual factors identified in this review were education level, atopy, and use of medication.

This review found several sociodemographic factors associated with OA. Lower education levels were significantly associated with chronic respiratory symptoms. A lower education level may delay treatment seeking among workers with respiratory symptoms. In a study conducted in Canada among the subjects diagnosed with OA, those with not more than primary education were associated with taking a longer time to visit a physician after the onset of work-related symptoms (OR = 0.45, 95% CI = 0.08–2.64). Their study concluded that those with lower education reportedly took a longer time to first visit a physician due to greater concerns about the socioeconomic impact [24]. However, this study was excluded from both the final review and Table 2 because it did not satisfy the inclusion and exclusion criteria of the current systematic review.

Although age and income were associated with OA, only a small number of studies were able to identify these factors [13]. Most studies that included these factors based on the working age groups and did not analyse age as an associated factor. However, one study that compared OA and non-OA found that the age of onset was significantly higher among those with OA (42.6 vs 20.7 years) [25]. These findings showed that OA maybe more pronounced in the working age group who have worked longer.

Another factor associated with OA was atopy, whereby work-related conjunctivitis was a predictor of Baker’s Asthma (BA) [11]. Conjunctivitis is one of the symptoms of atopy, in addition to rhinitis and dermatitis. One study looked at rhinitis as a precursor for OA and work exacerbated asthma (WEA). They found that sneezing with itching (78%) and rhinorrhea (70%) were more frequent in subjects with OA than in those with WEA (61%, p = 0.004 and 57%, p = 0.038, respectively) [26].

The role of individual factors in OA might differ according to population. However, the development of OA was undoubtedly not limited to exposures to respiratory inhalants. Thus, a more wholistic approach should be adopted in the identification and management of OA.

### Recommendation

It is important to have a policy to increase awareness and change of behaviour regarding health and safety among workers and organisations. This is because control measures and early intervention for OA have been more effective than late interventions. Employees should attend health safety courses to empower themselves towards complying with the standard health and safety measures [20]. Employers must provide a safe and conducive working environment, such as proper ventilation and PPE, as poor ventilation can affect the health of food industrial workers [19]. Additionally, particulate or bioaerosol detection device can be implemented by the employers to continuously assess their working condition [8]. Periodic medical surveillance should also be conducted to assess and detect any possible risk in developing OA [21]. The Human Resource Department should closely monitor their workers to identify those with risks and shift them to another department with no exposure, if they began to show symptoms. The use of PPE among workers should be emphasised, if exposure reduction methods are insufficient [27]. Lastly, cost benefit analysis must be conducted to ensure that the resources are efficiently used and able to prevent OA [28].

Characterising the complex interactions between work environment-related factors and individual factors is a crucial step in identifying the factors that determine the development of OA, and in implementing cost-effective prevention strategies. Prevention strategies can be applied to pre-employment and routine screening programmes to identify those at a higher risk of developing OA, while instilling occupational health education and tighter monitoring.

### Limitation

As with any research, this systematic review was not without limitations. The role of publication bias in this review must be acknowledged, as grey literature was not included. Furthermore, language bias should be considered, as only articles published in English were selected, although the search strategy in this review resulted in articles from several countries where English is not the primary language (e.g., France). Despite these limitations, this systematic review has successfully synthesised research evidence related to factors associated with OA among food industry workers. This review may serve as a guide towards improving service delivery strategies for preventing and controlling OA, especially among food industry workers who face considerable exposure to allergens.

## Conclusion

The management of occupational asthma is important for a better understanding of the development of this disease and the potential risk factors affecting a worker’s quality of life. With better knowledge of the determinants, it would be possible to implement preventative measures. Therefore, understanding the factors that are influencing the prevalence of OA, as highlighted in this review, is critical. These findings may be utilised to improve the implementation of OA prevention programmes, which would help to maximise their success.
